# Factors Associated with “Survivor Identity” in Men with Breast Cancer

**DOI:** 10.3390/curroncol28030158

**Published:** 2021-04-30

**Authors:** Kathryn L. Dalton, Sheila N. Garland, Peggy Miller, Bret Miller, Cheri Ambrose, Richard J. Wassersug

**Affiliations:** 1 Department of Psychology, Faculty of Science, Memorial University, St. John’s, NL A1B 3X9, Canada; kdalton@mun.ca (K.L.D.); Sheila.garland@mun.ca (S.N.G.); 2 Discipline of Oncology, Faculty of Medicine, Memorial University, St. John’s, NL A1B 3V6, Canada; 3 The Male Breast Cancer Coalition, Prairie Village, KS 66208, USA; peggy@malebreastcancercoalition.org (P.M.); bretmiller85@gmail.com (B.M.); cambrose0214@gmail.com (C.A.); 4 Department of Cellular & Physiological Science, Faculty of Medicine, University of British Columbia, Vancouver, BC V6T 1Z3, Canada

**Keywords:** male breast cancer, cancer survivor, cancer identity, sex, gender, survivorship

## Abstract

Cancer patients vary in their comfort with the label “survivor”. Here, we explore how comfortable males with breast cancer (BC) are about accepting the label cancer “survivor”. Separate univariate logistic regressions were performed to assess whether time since diagnosis, age, treatment status, and cancer stage were associated with comfort with the “survivor” label. Of the 70 males treated for BC who participated in the study, 58% moderately-to-strongly liked the term “survivor”, 26% were neutral, and 16% moderately-to-strongly disliked the term. Of the factors we explored, only a longer time since diagnosis was significantly associated with the men endorsing a survivor identity (OR = 1.02, *p* = 0.05). We discuss how our findings compare with literature reports on the comfort with the label “survivor” for women with BC and men with prostate cancer. Unlike males with prostate cancer, males with BC identify as “survivors” in line with women with BC. This suggests that survivor identity is more influenced by disease type and treatments received than with sex/gender identities.

## 1. Introduction

The National Cancer Institute (NCI) defines a “survivor” as one who remains alive and continues to function during, and after, overcoming a serious hardship or life-threatening disease [1]. Approximately 40 years ago, the American Cancer Society (ACS) declared that the term “survivor” could be applied to anyone diagnosed with cancer, regardless of treatments received or where they were in the treatment trajectory. With that broad definition, a person is considered a “survivor” from the time of diagnosis until the end of life [1].

How the term “survivor” is understood by patients may affect their quality of life and the care they are comfortable receiving. Endorsing the label or accepting the identity “survivor” has been associated with greater positive mood, satisfaction with life, and higher self-care [2,3]. However, not all people diagnosed with cancer are comfortable adopting the “survivor” label. Some individuals diagnosed with cancer may accept the label “survivor” even if they have not received curative treatment, whereas others, who may not have received aggressive treatments, may reject the label [4,5]. Individuals who hold the view that a “survivor” must fight a “battle”—accepting a military metaphor for dealing with cancer—may be reluctant to accept hospice care on the grounds that it is tantamount to “surrendering to the enemy” [6]. Such individuals may believe that to honorably and justifiably identify as a “survivor”, one must have fought a battle and overcome a serious hardship (more in line with the NCI’s definition of a survivor) [3,7,8]. This is particularly problematic since quality of life, and even longevity, can be enhanced for patients with advanced cancer if they are willing to accept early palliative care or hospice [9,10].

Researchers [5,7,11,12] and patient groups have recognized that the term “survivor” can be understood in a variety of ways within the oncology community and society at large [3,13,14]. Factors such as cancer type, treatment type, disease status, prior history of cancer, and sex/gender may influence the adoption of the label “survivor” [3,11,13,14]. Much of the research on “survivor” identify has been with females with breast cancer (BC) [2,4,9]. In a systematic review, over 77% of females treated for BC liked the label “survivor”; in contrast, only 30% of males with prostate cancer liked the label [11]. Prostate cancer patients ranked the lowest among patients diagnosed with a range of different types of cancer [11]. Other studies have come up with slightly different percentages that were influenced by the patients’ age and time since treatment (e.g., [3,14]). At one extreme, in a study with 200 males with prostate cancer and 200 females with BC, only 0.5% of the prostate cancer patients liked the label “survivor”, and 22% of the females with BC liked the label [15]. Unfortunately, males with breast cancer were not included in any of the studies that we know of. As such, we do not know if males treated for BC would be more similar in their comfort with the label “survivor” compared to individuals with whom they share a disease (i.e., women), or more in line with the prostate patients with whom they share the same lived experience of sex and gender [16,17]. 

Breast and prostate cancer have both similarities and differences in disease treatment and progression. Because of screening, both breast and prostate cancers can be identified before they are largely symptomatic [13,18]. The mean age for diagnosis is similar, i.e., 62 for females with BC breast cancer and ~65 for prostate cancer [19,20]. The ten-year survival for both disease is similarly high at 84% for invasive female BC and 99% for prostate cancer [21], though lower for males with BC at 71%) [19]. However, treatment progress can differ substantially between the two diseases [22]. Those diagnosed with BC often receive multimodal treatments—i.e., chemotherapy, surgery, and radiation—over a relatively short period with the potential for a cure. In contrast, if a prostate cancer patient is diagnosed with non-metastatic disease, he receives either surgery or radiation with curative intent [22] and is typically not offered chemotherapy at that point, since chemotherapy for prostate cancer is palliative and not curative. Thus, prostate cancer treatments are often spread out over a longer period than BC treatments, with different treatment modalities introduced incrementally as the disease progresses. Furthermore, men with prostate cancer can expect to get prostate-specific antigen tests on a regular basis for the rest of their lives [23]. The pattern of protracted disease progression with incremental treatment [23] and lifelong PSA screening may account in part for why men with prostate cancer are more inclined to view themselves as “patients” rather than “survivors” [12].

To date, there have been no studies assessing comfort with the “survivor” label for males with BC. The purpose of this study was to examine how comfortable males with BC are with accepting the label cancer “survivor” and examine factors associated with acceptance of “survivor” identity. 

## 2. Materials and Methods

The study was approved by Memorial University’s Health Research Ethics Board (No: # 2019.249) and all participants provided informed consent. This research was designed with input from male BC advocates affiliated with a community support group for males with BC. Males with a BC diagnosis were invited to participate in an online cross-sectional survey about their comfort with the label cancer “survivor” versus other terms that have been applied to individuals diagnosed and treated for a disease. All data were anonymously collected via Qualtrics survey software posted from January to April 2020. IP addresses were recorded to ensure the survey could not be completed twice from a single internet address. Completion of the survey took approximately 10 min, and no compensation was provided. 

### 2.1. Participants and Recruitment

Eligibility criteria for this study were intentionally broad in order to capture a diversity of experiences. To be eligible, participants were required to be biologically male, able to understand and read English, and must have received a BC diagnosis. Recruitment was primarily through the social media platforms of community support and advocacy groups in the USA and Australia. We also asked those who completed the survey to bring it to the attention of any other males with BC they knew (i.e., snowball recruiting) and to pass the link to the survey on to their health care providers as well. Further, we used PubMed to identify medical researchers who have published on male BC in the last five years and invited them to share the survey with their male BC patients. Lastly, we used our personal social media (i.e., Twitter) accounts to invite participation with relevant hashtags. 

### 2.2. Measures

The survey included questions on basic demographics and BC treatment history. The primary objective, how males with BC felt about the label cancer “survivor”, was assessed using three “survivor” identity questions used in previous studies [15]. First, participants were asked to rate their approval of each of the following labels: patient, client, consumer, survivor, partner, and customer. A five-point Likert scale was used with options ranging from “strongly dislike” to “strongly like”. Participants were then asked to rate how much they personally felt like each of the following terms described them: victim of cancer, cancer patient, person who has had cancer, cancer survivor. A five-point Likert scale was used, with responses ranging from “not at all” to “very much”. For the third question, participants were asked to choose which label they felt best describes them with the same options as the second question (victim of cancer, cancer patient, person who has had cancer, cancer survivor, and other). Participants could only select one option.

The survey also involved a series of open-ended questions based on participants’ endorsement of “survivor” identity. The open-ended questions explored why the men considered themselves a “survivor” or not. If they endorsed feeling like a “survivor”, they were asked when this began, and if they did not endorse feeling like a “survivor” we asked them to explain why not. Lastly, as a general exploratory question, we asked all participants what they thought the difference was between a BC “survivor” and a BC “patient,” and if they felt the experience of BC is different for males as opposed to females.

### 2.3. Analyses

Frequencies were tabulated for categorical survivor identity questions. The rate of missing data was considered to be low. Overall, there were 3% missing data on the three questions about identity, ranging from 1.4% to 5.7% of responses on the 3 individual questions [24]. The open-ended questions were content analyzed with individual responses reviewed and common statements grouped into themes. 

Separate univariate logistic regressions were performed to assess whether the prespecified factors of time since diagnosis, age, currently on treatment, and cancer stage were associated with comfort with the “survivor” label. Given the sample size constraints, these variables were chosen based on previous literature suggesting possible associations with “survivor” identity. Logistic regression uses complete case analysis and can provide asymptotically unbiased estimates under a wide range of missing-data assumptions [25]. Statistical significance was set at 0.05.

## 3. Results

The demographic characteristics of the men (*N* = 70) who participated are presented in Table 1 below. The participants were 63 years old on average (range 34–84). Ninety four percent reported their race as White. The majority were from the USA (45%) or Australia (21%), and a one-way ANOVA revealed that the populations did not differ on any parameters, and therefore, they were merged. The most common cancer stage was one (33%) or two (39%), and the majority were currently receiving treatments (59%). Table 2 below reports clinical characteristics of the sample.

### 3.1. How Males with Breast Cancer Feel about “Survivor” Identity

**Figure 1:**
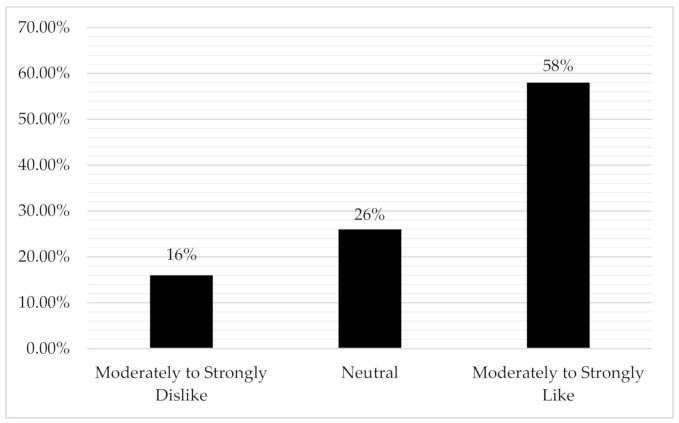
Evaluation of preference for “survivor” label in males with breast cancer.

Concerning “survivor” identity, 58% of the males with BC moderately-to-strongly liked the term “survivor”, 26% were neutral, and 16% moderately-to-strongly disliked the term (Figure 1). Forty-four percent believed the term “cancer survivor” best describes their identity compared to “a person who has had cancer” (36%), a cancer “patient” (14%), “victim” (1.5%), and “other” (4.5%). 

Participants reported that they were more comfortable endorsing the “survivor” label if they were cancer-free without reoccurrence, had finished all cancer treatments, and they felt like they were “still living” and/or “back to normal”. For example, one participant stated, “*[I am a survivor] because I am able to live my life as fully as before being diagnosed; I feel like a breast cancer survivor*”. Males were not as comfortable endorsing the “survivor” label if they still experienced significant fear of recurrence or felt that they did not want to be defined by cancer.

When asked how BC is different for males compared to females, males with BC reported that although their cancer is treated similarly, they are viewed differently than females with BC. Specifically, men reported that there is less research, support, and awareness of male BC, and as a result, some men said they have felt alone, stigmatized, and not understood. Conversely, other men stated that they felt BC is *easier* for men than women because loss of breasts/hair/etc. are beauty/cosmetic/self-image components that carry more meaning for women than for men. One participant stated:

“*A lot of female identity since puberty is associated with the breasts, from both a beauty aspect and a potentially nurturing, mothering role. Losing one or both breasts is potentially very destructive to a woman’s sense of self and her role in life. Men don’t share this level of identification, but the scar remaining from a male mastectomy can be disfiguring and a source of embarrassment at, for example, the beach, if one allows it to be*”.

Another participant said:

“*Breast cancer is still cancer, whether viewed as traditionally a woman’s disease or not, so the diagnosis, treatment and follow up is exactly the same. In my personal situation, I did not experience any loss of identity that women may experience, only because my physical appearance did not change*”.

### 3.2. Factors Associated with Endorsing “Survivor” Identity in Males with BC

Separate univariate binomial logistic regressions models were used to identify significant independent factors associated with endorsing survivor identity (see Table 3 below). There was a positive relationship between greater months since diagnosis and endorsement of “survivor” identity (OR = 1.02 [1.00, 1.04] *p =* 0.050). However, endorsing a “survivor” identity was not significantly associated with age, treatment status, or cancer stage.

## 4. Discussion

This is the first study to examine how males diagnosed with and treated for BC feel about the label cancer “survivor”. We found that 58% of males with BC moderately-to-strongly liked the term “survivor”, compared to only 16% moderately-to-strongly disliking the term. Longer time since diagnosis was associated with a greater likelihood of endorsing a survivor identity. Previous researchers have also reported that longer time since diagnosis and treatment is correlated with increased comfort with the label survivor for other cancer populations [3].

Of the various factors we examined, the only one associated with a higher likelihood of endorsing survivor identity was greater time since diagnosis. This fits with the simple and unsurprising fact that comfort with the label “survivor” grows the longer an individual treated for cancer actually survives [3] (despite a definition of survivor that presumes one is a “survivor” from the time of diagnosis). Conversely, being referred to as a survivor may also alienate those with a cancer whose prognosis is not as favorable. For example, pancreatic, liver, and lung cancer all have five-year survival rates of <20% [26]. As such, few individuals diagnosed with one of those diseases live long enough to relish the label “survivor”. This may leave certain individuals unable to identify with others or organizations, who arbitrarily label them as survivors even when though they are not comfortable or confident with that label. Many supportive care programs and service for individuals treated for a cancer have the word “survivor” in their name. Examples include the Canadian Cancer Survivor Network, the National Coalition for Cancer Survivorship, and the American Cancer Society Cancer Survivors Network^®^ (CSN). It has yet to be investigated whether an organization’s name itself discourages participation by patients, who are not personally comfortable with the label “survivor”. 

Our findings may also be relevant to the development of “survivorship care plans” and the transition of patients from the oncological setting back to management by primary care physicians. Much has been written about how to make this transition smooth and reassuring for patients [27]. Our research suggests that the patients’ comfort with the transition could be influenced by the fact the documentation associated with the transfer of care includes the word “survivor[ship]”. Although this has not been investigated, we hypothesize that a patient who is not comfortable with the label “survivor” may similarly be resistant to accepting a “survivorship care plan” based simply on the document’s name.

Comparing males with BC to females with BC and males with prostate cancer may provide insight into the extent to which comfort with the label “survivor” is influenced by sex/gender difference between men and women versus the specific cancers and the ways they are diagnosed, managed, and progress. In our sample, 78% of the males with BC reported that they moderately or strongly liked the term “survivor”. In contrast, in the Deber et al. [15] study, 22% of females with BC and 0.5% of the males with prostate cancer liked the term “survivor”. Considering that comfort with the label cancer “survivor” is higher for both men and women treated for BC, this suggests that the label “survivor” may be accepted because of the nature of the disease and its treatments. If males with BC had shown less comfort with the term than females with BC, and findings were more similar with what has been reported for prostate cancer patients, this would suggest that male gender norms may influence men’s acceptance of and comfort with the label “survivor” [28,29]. As such, it appears that acceptance of the label “survivor” for individuals diagnosed and treated for a cancer influenced more by the disease itself than by sex or gender stereotypes. Our results also show that comfort with the label “survivor” varies greatly as noted by several previous authors [4,7,11]. 

Differences in diagnosis and prognosis for BC may explain why males with BC reported higher endorsement of survivor identity than females. BC is typically diagnosed at a later stage in men than in women [21]. This may be due to the fact that men are more likely than women to not recognize signs of the disease or ignore them and thus fail to seek medical attention in a timely fashion [30]. Since BC is commonly more advanced when diagnosed in men, it is subsequently more fatal. Based on 2018 US estimates, 19% of males with BC do not survive, compared to 15% of female BC patients [16]. This could contribute to, but not fully explain, why males with BC may be more likely to endorse the “survivor” label than females with the same disease. Given the greater risk of death for men, it is not surprising that they might endorse the label “survivor” more than women with BC. 

The dislike for the label “survivor” for men treated for prostate cancer compared to males with BC may seem surprising given prostate cancer’s high survival rate; i.e., over 99% and >85% at five years [21]. One potential explanation may relate to the time course and sequencing of treatments for prostate cancer versus BC. Because of slow disease progression, prostate cancer patients may have no sign of residual disease after primary treatment yet experience a subsequent rise in their prostate-specific antigen (PSA) test months to years later. Even with stable PSA, men can expect to have their cancer continually monitored, leaving them in the mindset of a person with a chronic disease rather than an individual who is now disease-free (i.e., a “survivor”). For prostate cancer patients, the number of treatment options that are not curative but can extend life by a year or more continues to grow. The continual monitoring and treatment of prostate cancer may solidify their identity as a patient rather than as a “survivor”.

Our study has a number of limitations. Notably, our sample size is small. However, considering that less than 1% of all BC diagnoses are in men [16,31], recruiting a large sample from that population is always challenging. Our main method of recruitment was via organizations that provide information and support to men with BC, and this may have resulted in a sample of males with different characteristics or needs than males who would not be connected with these organizations. Further, we did not recruit comparable samples of women with BC or men with prostate cancer to be able to definitively compare differences, and although we used the same questions as Deber et al. [15], that study is 15 years old, and popular understanding and acceptance of the label cancer “survivor” may have shifted. However, to the best of our knowledge, that remains a definitive study on comfort with the label survivor for a variety of cancer patients. Furthermore, survival rates for BC and prostate cancer over the past 15 years have actually improved in a similar fashion for both diseases, further strengthening our results. The limited variation or race and ethnicity of the sample also prevented us from understanding the experiences of males with BC from other demographics.

## 5. Conclusions

Our data suggest that males with BC are comfortable with the label “cancer “survivor” and that comfort with the label “survivor” appears to be more influenced by disease type and treatment progression than with sex and gender stereotypes. It is important for oncology health care providers, cancer organizations, researchers, and patient groups to be aware that cancer “survivor” is not an identity that all individuals are comfortable with. Understanding the preferred label of individuals diagnosed with cancer is an important way to further support their care. 

## Figures and Tables

**Table 1:** Demographics.

Demographics	*n*	%
Age (range 34–84, mean = 63, SD = 10)		
Under 50	7	10
50–59	16	22.9
60–69	28	40
70–79	17	24.3
80+	2	2.9
Recruited from		
The Male Breast Cancer Coalition	42	60
Breast Cancer Network Australia	10	14.3
Health Care Professional	1	1.4
Social media (Twitter, Facebook)	6	8.6
Friend	4	5.7
Other	7	10
Country of Residence		
USA	32	45
Canada	2	2.9
Australia	15	21.4
Germany	5	7.1
Austria	1	1.4
Missing	15	21.4
Relationship Status		
Single/not in a relationship	6	8.6
In a committed relationship/married	64	91.4
Number of Children		
None	11	15.7
One	11	15.7
Two	34	48.6
Three or greater	14	20
Race/Ethnicity		
White	66	94.3
Black	1	1.4
Latin American	2	2.9
Other	1	1.4
Education		
High school degree or equivalent/Some college	30	42.9
Bachelor’s degree or Associate degree	26	37.1
Graduate or Professional degree	14	20
Currently Employed		
Yes	28	40
No	41	58.6
Missing	1	1.4
Annual Income		
Under $49,000 USD	25	35.7
$50,000 to 99,999 USD	29	41.4
$100,000 and greater USD	14	20.0
Missing	2	2.9

**Table 2:** Clinical characteristics of study participants.

Clinical Characteristics	*n*	%
Self-Reported Health Status		
Excellent	7	10
Very Good	28	40
Good	27	38.6
Fair	6	8.6
Poor	2	2.9
Diagnosis Date		
2004 or earlier	5	7.2
2005–2009	11	15.7
2010–2014	26	37.1
2015–2020	28	40
Stage ^a^		
0	3	4.3
1	23	32.9
2	27	38.6
3	12	17.1
4	2	2.9
Missing	3	4.3
Family History of Cancer		
Yes	50	71.4
No	20	28.6
Currently Receiving Treatment		
Yes	41	58.6
No	28	40
Missing	1	1.4
Treatment History		
Surgery		
Past	60	85.7
None	10	14.3
Chemotherapy		
Current	6	8.6
Past	39	55.7
None	25	35.7
Radiation		
Current	3	4.3
Past	32	45.7
None	35	50
Hormonal		
Current	35	50
Past	19	27.1
None	16	22.9
Immunotherapy		
Current	2	2.9
Past	2	2.9
None	66	94.3
Co-morbid Illnesses		
0–1	35	50
2–3	27	38.6
3+	7	10
Missing	1	1.4

^a^ We asked the participants their cancer stage at diagnosis in an open-ended question, and some participants gave us a description of their disease’s aggressiveness at diagnosis rather than simply the number on the standard 1–4 scale. There were thirteen such entries that required conversion to standard stages. Three entries were either too imprecise to convert to a standard stage or the participant simply stated that they did not know the stage.

**Table 3:** Binomial logistic regression examining variables associated with endorsing survivor identity.

Univariate Logistic Regression Analysis		
Parameter	Odds Ratio (95% CI)	*p*
**Age**	1.045 (0.980, 1.11)	0.177
**Stage**		
Early (0–2)	reference	
Late (3–4)	1.35 (0.246, 7.42)	0.730
**Currently on Treatment**		
Yes	reference	
No	2.81 (0.647, 12.18)	0.168
**Months since Diagnosis**	1.02 (1.00, 1.04)	0.050

## Data Availability

Data will not be deposited in a repository. Interested researchers are encouraged to contact the corresponding author for data sharing requests.
